# Knowledge, attitudes, practice and barriers of physicians to provide tobacco dependence treatment: a cluster analysis

**DOI:** 10.11604/pamj.2021.38.193.27047

**Published:** 2021-02-19

**Authors:** Moon Fai Chan, Yaqoub Alsaidi, Sana Al-Sumri, Buthaina Al-Maskari, Hajer Al-Hamrashdi

**Affiliations:** 1 Department of Family Medicine and Public Health, Sultan Qaboos University, Muscat, Oman,; 2 Medical Services Royal Guard of Oman, Muscat, Oman

**Keywords:** Primary care physician, Oman, cluster analysis, knowledge, attitudes, practice, barriers

## Abstract

**Introduction:**

in Oman, there is a need to understand the profile of primary care physicians’ (PCP) knowledge, attitude, and practice, and barriers (KAPB) towards tobacco dependence treatment (TDT). Their profile will directly affect their consultation and contribution to health care educators to develop an appropriate educational program for the PCPs. The aim of this study is to determine profiles in a cohort of PCP with regards to factors associated with physicians’ perceived KAPB of providing TDT.

**Methods:**

a cross-sectional survey was conducted for four months from September to December 2019. A sample of 226 (response rate is 71.2%) PCPs working for Muscat's health centers, the capital of Oman, was collected. A 2-step cluster method was used to separate the sample into sub-groups according to their demographic and KAPB scores.

**Results:**

cluster analysis revealed two groups of PCPs who are different in demographics and KAPB scores. The PCPs in cluster B (27.4%) have higher educational levels, senior ranking, more males and older. They labeled as the “good knowledge, positive attitudes, and highly practices” group. The PCPs in cluster A comprised 72.6% of our samples. There are more females, younger, and with a junior ranking. This cluster was identified as the “lack of knowledge, moderate attitudes, and rarely practices” group.

**Conclusion:**

findings might help primary health care authorities to address this preventable issue and plan interventions to establish well-structured TDT clinics in the future.

## Introduction

There are about 1.3 billion tobacco users in the world, and about 6 million people die each year from tobacco use [1]. The World Health Organization (WHO) reported that adult smoking in the Arabic countries, the prevalence rate on smoking among men and women was 14.2% and 0.6%, respectively [2]. In the Gulf Cooperation Council (GCC) countries, the highest prevalence rates on smoking are in Kuwait (male: 40.5%, female: 4.4%), and followed by United Arab Emirates (male: 34.9%, female: 4.2%), and the third one is Bahrain (male: 33.2%, female: 6.8%) [3].

It is well-known that smoking will induce heart disease, high blood pressure, and lung cancer [4]. Quit smoking is hard for smokers if a lack of advice and supports were received [5]. A systematic review showed that giving a brief and intensive advised by a health practitioner had a 66% and 84%, respectively, chance of quitting smoking [6]. Another study suggested that healthcare professionals who had acquired training have been much more likely to carry out smoking cessation duties than untrained doctors [7]. Nowadays, one of the most effective ways to help smokers quit smoking is tobacco-dependent treatment (TDT) [6]. In Oman, three smoking cessation clinics provided the TDT to smokers who want to quit smoking. Although the clinics offered both nicotine replacement therapy (NRT) and psychotherapy, experienced and well-trained physicians to provide appropriate counseling to the patients are important. A study in Arabic countries found that primary healthcare providers had poor knowledge of smoking cessation counseling, and their practices are unsatisfactory [8]. Another study found that smoking cessation is determined by the level of knowledge and skills of the primary care professions [9].

To the best of the author´s knowledge, no previous studies were conducted in Oman to identify the knowledge, attitudes, practices, and barriers (KAPB) of primary health care physicians (PCP) towards providing the tobacco dependence treatment (TDT) service. Hence, this study aimed to explore any different profiles exist in a cohort of PCPs with their demographic and KAPB of providing TDT. We hypothesised that, if the PCPs had a different type of profiles on demographic and KAPB levels, then understanding each each profile group is important for healthcare educators to develop an integrated health education to promote TDT. So, two specific research objectives were formulated: 1) to identify the demographic and perceived KAPB of providing TDT profiles of PCPs; and 2) to explore different patterns of PCPs in terms of demographic and KAPB of providing TDT on different groups.

## Methods

**Design**: this survey was conducted to all health centers in Muscat between September 2019 to December 2019, utilizing data from a previous study conducted by the same research team [10]. This is a sub-group analysis because cluster analysis required completed data for analysis. Therefore, only 226 completed records were used in this study.

**Subject**: the PCPs in health centers will be non-certified general practitioners, family medicine residents, or certified family physicians. According to a national report from the Ministry of Health on primary care in Muscat governorate in 2018, 432 physicians worked in the primary health care institutions. There are around 313 PCPs are working in the health centers in Muscat governorate. The power analysis is based on the previous study conducted by Al-Jdani and her colleagues [8]. This study expected a margin of error of 0.03 and a confidence interval level of 95%, calculated by nQuery Advisor, the required sample size of this study would be 243 [11].

**Instrument**: the instrument consisted of two sections: Section A collected PCPs demographic data (e.g., age, gender, current position). Section B comprised questions on the KAPB of providing TDT [8] and consisted of 4 parts. Part 1 consists of 12 multiple-choice questions (MCQ) that obtained PCP´s knowledge of providing TDT. One point for each question and the total scores were ranged from 0 to 12, with higher scores indicated a higher-knowledge. The items are in dichotomous data, so we used Kuder-Richardson 20 (KR20) coefficient and its internal consistency is 0.642; Part 2 is attitudes questions, which consisted of 7 questions that each was ranked from Strongly Disagree (1) to Strongly Agree (5). The higher scores indicate stronger agreement on attitudes, and its Cronbach alpha is 0.642; Part 3 comprised practice questions consisted of 10 items, each ranked on a four-point scale, from Never (0) to Always (3). Higher scores indicated more frequently in providing TDT. Its Cronbach alpha is 0.873; Part 4 had a total of 5 MCQs (Yes or No per question) that asked for what kind of barriers that PCPs experienced when providing TDT, and the KR20 is 0.53.

**Data collection and ethical issues**: ethical approval was obtained from the local research ethics committee (Ref: MOH-DGPS-MG-46/2019). Approval to use the KAP questionnaire has been obtained from the author. PCPs from all the health centers in Muscat governorate were contacted and invited to participate in the study. To ensure a high response rate, the research team had contacted the head of each health center. They help explain to their staff the purpose of the study and how to fill in the questionnaire. A cover letter and a sealed envelope were provided to ensure confidentiality. All of the participants signed written informed consent. The research team sent a reminder to the heads to instruct their staff to complete their questionnaire and submit them within three weeks. The research team collected the questionnaires from the head of each health center. Neither staff name nor ID was collected from the returned questionnaire to ensure privacy and confidentiality.

**Data analysis**: the statistical package for the social sciences (IBM SPSS 23.0) was used for data analysis. To address objective 1, a 2-step cluster method was used to separate the sample into sub-groups, if both categorical and numerical variables were included in the study sample [12]. This method is the most appropriate way to use if we suspect the sample’s heterogeneity. The analysis will include two main variables: (1) demographic and (2) KAPB factors. The choice of a similarity measure was determined by minimizing the change of Schwarz´s Bayesian Criterion (BIC) values [13]. To address objective 2, when the samples had been identified into clusters, group comparison was performed. χ^2^ / Fisher´s exact tests were used to examine any significant associations among clusters for categorical data. For numerical data, independent t-test was used to examine any significant differences among clusters. Partial correlation was used to quantify the relationships among knowledge, attitudes, and practice scores by clusters. All tests will set P<0.05 as a level of significance.

**Ethical consideration**: ethical approval was obtained (Ref: MOH-DGPS-MG-46/2019).

## Results

**Cluster analysis**: the cluster method yielded two clusters based on BIC change (ratio = 2.65). In clusters A and B, there were 164 (72.6%) and 62 (27.4%) PCPs, respectively. The two clusters were compared with demographic and KAPB outcomes.

**Demographic factors**: as shown in Table 1, the cluster analysis revealed two groups. Cluster A was characterized by more females (n=146, 89.0%), relatively younger in age (Mean±SD = 33.2±5.9 years), the majority are GP (n=133, 81.1%) and didn´t received training on smoking-cessation (n=136, 89.0%). They received information on TDT are majority through their undergraduate study (n=103, 62.8%) and internet (n=101, 61.6%). The PCP in Cluster B are older (37.0±6.5); the majority are specialist in family medicine (n=52, 71.0%) and had received training on smoking-cessation (n=44, 71.0%). They received information on TDT are majority through their postgraduate study (n=42, 67.7%), conducted research (n=32, 51.6%), and attended workshop (n=28, 45.2%). Compared with the PCPs in clusters A and B, significant differences were found in gender (p=.001), current position (p<.001), age (p<.001), received training on smoking-cessation (p<.001), and all sources of information on TDT except through colleagues (p=0.189) and reading textbook (p=0.238).

**Table 1:** comparison of demographic of the study sample by cluster

		Cluster			
	Total (n=226)	A (n=164, 72.6%)	B (n=62, 27.4%)		
**Demographic**	n (%)	n (%)	n (%)	Statisticsa	p-value
**Gender**					
Male	36 (15.9)	18 (11.0)	18 (29.0	10.954	0.001
Female	190 (84.1)	146 (89.0)	44 (71.0)		
**Current position**					
GP	143 (63.3)	133 (81.1)	10 (16.1)	104.811	<.001
Family medicine resident	34 (15.0)	22 (13.4)	12 (19.4)		
Family medicine Specialist	29 (12.8)	7 (4.3)	22 (35.5)		
Family medicine Senior Specialist	16 (7.1)	2 (1.2)	14 (22.6)		
Family medicine senior consultant	4 (1.8)	0 (0.0)	4 (6.5)		
Age (Years) Mean ± SD	34.3 ± 6.3	33.2 ± 5.9	37.0 ± 6.5	4.135b	<.001
**See smoker patients per week**					
Don't know	47 (20.8)	37 (22.6)	10 (16.1)	5.927	0.115
< 5 patients / week	109 (48.2)	77 (47.0)	32 (51.6)		
5-10 patients / week	42 (18.6)	34 (20.7)	8 (12.9)		
10+ patients / week	28 (12.4)	16 (9.8)	12 (19.4)		
**Received training on smoking-cessation**					
No	164 (72.6)	146 (89.0)	18 (29.0)	81.339	<.001
Yes	62 (27.4)	18 (11.0)	44 (71.0)		
**Source of information - undergraduate**					
No	95 (42.0)	61 (37.2)	34 (54.8)	5.748	0.017
Yes	131 (58.0)	103 (62.8)	28 (45.2)		
**Source of information - Postgraduate**					
No	136 (60.2)	116 (70.7)	20 (32.3)	27.79	<.001
Yes	90 (39.8)	48 (29.3)	42 (67.7)		
**Source of information - colleagues**					
No	145 (64.2)	101 (61.6)	44 (71.0)	1.722	0.189
Yes	81 (35.8)	63 (38.4)	18 (29.0)		
**Source of information - Textbook**					
No	138 (61.1)	104 (63.4)	34 (54.8)	1.392	0.238
Yes	88 (38.9)	60 (36.6)	28 (45.2)		
**Source of information - Research**					
No	160 (70.8)	130 (79.3)	30 (48.4)	20.752	<.001
Yes	66 (29.2)	34 (20.7)	32 (51.6)		
**Source of information - the Internet**					
No	97 (42.9)	63 (38.4)	34 (54.8)	4.954	0.026
Yes	129 (57.1)	101 (61.6)	28 (45.2)		
**Source of information - Conference**					
No	198 (87.6)	154 (93.9)	44 (71.0)	21.803	<.001
Yes	28 (12.4)	10 (6.1)	18 (29.0)		
**Source of information - Workshop**					
No	183 (81.0)	149 (81.4)	34 (54.8)	37.878	<.001
Yes	43 (19.0)	15 (9.1)	28 (45.2)		

**a**, chi-square test; **b**, independent t-test

**Knowledge, attitude, practice, and barrier factors**: from Table 2 and Table 3, on the knowledge items, almost all the PCPs in cluster A were reported fewer correct answers than PCPs in cluster B in all items. The PCPs in cluster B (7.29±1.72) had a higher knowledge score than PCPs in cluster A (3.76±1.68; t=14.013, p<.001). On the attitudes scores, PCPs in cluster B (27.42±4.77) reported a more positive attitude toward providing TDT than PCPs in cluster A (25.02±2.97; t=3.692, p<.001). On the practice items, almost all the PCPs in cluster A were reported fewer practices of providing TDT than PCPs in cluster B in all 10 items. The PCPs in cluster B (21.48±4.78) had a higher practice score than PCPs in cluster A (15.18±4.95; t=8.626, p<.001). In Table 3, on the barriers of providing TDT items, the PCPs in cluster B had reported more barriers to perform TDT than PCPs in cluster A, especially on the failure of follow up 77.4% vs. 40.2%, p<.001), relapse and withdrawal symptoms (58.1% vs. 29.9%, p<.001), and patient desired to change (71.0% vs. 48.8%, p=.0003). Also, more PCPs in cluster B than cluster A suggested to add tobacco status as a mandatory field in the health information system (83.9% vs. 70.1%, p=0.036), and to add tobacco status to the vital signs will encourage the physicians to provide TDT service (83.9% vs. 61.0%, p=0.001).

**Table 2:** comparison of knowledge, attitudes, and practice of the study sample on tobacco dependence treatment by cluster

		Cluster			
	Total (n=226)	A (n=164, 72.6%)	B (n=62, 27.4%)		
KAP on tobacco dependence treatment	n (%)	n (%)	n (%)	Statisticsa	p-value
**Knowledge (only show Correct answer)**					
First-line pharmacological agent	202 (89.4)	142 (70.3)	60 (29.7)	4.921	0.027
The method for TDT short and long term success	186 (82.3)	132 (80.5)	54 (29.0)	1.349	0.245
Familiar of 5As	102 (45.1)	42 (41.2)	60 (58.8)	92.013	<.001
Components of 5As	110 (48.7)	52 (31.7)	58 (93.5)	68.873	<.001
NRT has especial importance in specific patient	93 (41.2)	53 (32.3)	40 (64.5)	19.262	<.001
TDT relapse rate is high	74 (32.7)	48 (29.3)	26 (41.9)	3.278	0.07
Depressed mood is one of nicotine withdrawal symptoms	84 (37.2)	58 (35.4)	26 (41.9)	0.831	0.362
The fastest NRT method	55 (24.3)	29 (17.7)	26 (41.9)	14.371	<.001
Hiccups are common side effect of nicotine gum	16 (7.1)	6 (3.7)	10 (16.1)	10.636b	0.002
Sleep can be affected by nicotine patches	48 (21.2)	18 (11.0)	30 (48.4)	37.644	<.001
Nicotine patches can be used with bupropion	42 (18.6)	16 (9.8)	26 (41.9)	30.792	<.001
Varenicline is as effective as sustained release bupropion	56 (24.8)	20 (12.2)	36 (58.1)	50.787	<.001
**Total score (Mean ± SD)**	**4.73 ± 2.31**	**3.76 ± 1.68**	**7.29 ± 1.72**	**14.013c**	**<.001**
**Attitudes**					
**Q1. Performing smoking cessation counseling for my patients is important**					
Strongly Disagree / Disagree	220 (97.3)	160 (97.6)	60 (96.8)	6.805c	0.031
Neutral	4 (1.8)	4 (2.4)	0 (0.0)		
Strongly Agree / Agree	2 (0.9)	0 (0.0)	2 (3.2)		
**Q2. I feel confident in my abilities to perform smoking cessation counseling for my patients**.					
Strongly Disagree / Disagree	67 (29.6)	35 (21.3)	32 (51.6)	24.616	<.001
Neutral	95 (42.0)	71 (43.3)	24 (38.7)		
Strongly Agree / Agree	64 (28.3)	58 (35.4)	6 (9.7)		
**Q3. I should routinely ask about my patients’ smoking habits**.					
Strongly Disagree / Disagree	187 (82.7)	135 (82.3)	52 (83.9)	0.670c	0.679
Neutral	34 (15.0)	26 (15.9)	8 (12.9)		
Strongly Agree / Agree	5 (2.2)	3 (1.8)	2 (3.2)		
**Q4. I should routinely advise my patients to quit smoking**.					
Strongly Disagree / Disagree	206 (91.2)	146 (89.0)	60 (96.8)	7.368c	0.019
Neutral	16 (7.1)	16 (9.8)	0 (0.0)		
Strongly Agree / Agree	4 (1.8)	2 (1.2)	2 (3.2)		
**Q5. If I advise my patients to quit continuously, their chances of quitting smoking are increased**.					
Strongly Disagree / Disagree	153 (67.7)	109 (66.5)	44 (71.0)	0.655	0.721
Neutral	45 (19.9)	33 (20.1)	12 (19.4)		
Strongly Agree / Agree	28 (12.4)	22 (13.4)	6 (9.7)		
**Q6. If I'm an active smoker, I will be less likely to advise my patients to stop smoking**.					
Strongly disagree / disagree	76 (33.6)	58 (35.4)	18 (29.0)	4.004	0.135
Neutral	78 (34.5)	60 (36.6)	18 (29.0)		
Strongly agree / agree	72 (31.9)	46 (28.0)	26 (41.9)		
**Q7. I am satisfied with my knowledge and skills in smoking cessation counseling**.					
Strongly disagree / disagree	28 (12.4)	6 (3.7)	22 (35.5)	45.518	<.001
Neutral	62 (27.4)	44 (26.8)	18 (29.0)		
Strongly agree / agree	136 (60.2)	114 (69.5)	22 (35.5)		
**Total score (Mean ± SD)**	**25.68 ± 3.70**	**25.0 ± 2.97**	**27.4 ± 4.77**	**3.692c**	**<.001**
**Practice (only show always/sometimes respondents)**					
Ask	202 (89.4)	140 (85.4)	62 (100.0)	10.151	<.001
Document	197 (87.2)	137 (83.5)	60 (96.8)	7.049	0.008
Update	114 (50.4)	70 (42.7)	44 (71.0)	14.399	<.001
Explain	199 (88.1)	139 (84.8)	60 (96.8)	6.177	0.013
Encourage	203 (89.8)	141 (86.0)	62 (100.0)	9.68	0.002
Teach	122 (54.0)	68 (41.5)	54 (87.1)	37.715	<.001
Discuss	126 (55.8)	78 (47.6)	48 (77.4)	16.259	<.001
Plan	75 (33.2)	31 (18.9)	44 (71.0)	55.005	<.001
Use pharmacological aid	46 (20.4)	22 (13.4)	24 (38.7)	17.758	<.001
Follow up	66 (29.2)	30 (18.3)	36 (58.1)	34.422	<.001
**Total score (Mean ± SD)**	**16.9 ± 5.65**	**15.1 ± 4.95**	**21.4 ± 4.78**	**8.626c**	**<.001**

**a:**chi-square test; **b:** Fisher's Exact test; **c:** Exact test; Knowledge, correct answer get 1 point, 12 items, total range from 0 to 12, higher scores indicated a higher-knowledge level; Attitudes: each item is from strongly agree (5) to strongly disagree (1), range from 7 to 35, a higher score indicates stronger agreement on positive attitudes; Practice, each item score from Always (3) to never (0), range from 0 to 30, a higher score indicated more frequently of providing TDT.

**Table 3:** comparison of barriers and encourage factors of the study sample on tobacco dependence treatment by cluster

		Cluster			
	Total (n=226)	A (n=164, 72.6%)	B (n=62, 27.4%)		
Factors	n (%)	n(%)	n (%)	Statisticsa	p-value
Barrier (only show Yes respondents)					
Physician lack of training	188 (83.2)	152 (92.7)	36 (58.1)	38.549	<.001
Physician lack of time	163 (72.1)	119 (72.6)	44 (71.0)	0.057	0.812
Failure of follow-up with the patients	114 (50.4)	66 (40.2)	48 (77.4)	24.873	<.001
Relapse and withdrawal symptoms	85 (37.6)	49 (29.9)	36 (58.1)	15.233	<.001
Patient undesired to change	124 (54.9)	80 (48.8)	44 (71.0)	8.944	0.003
Encourage (only show Yes respondents)					
Q1. Add tobacco status as a mandatory item (implemented in electronic medical records) to be filled by physicians in each visit.	167 (73.9)	115 (70.1)	52 (83.9)	4.409	0.036
Q2. Add tobacco status as a vital sign (implemented in electronic medical records) to be filled by physicians in each visit.	152 (67.3)	100 (61.0)	52 (83.9)	10.709	0.001
Q3. Add tobacco status as mandatory (implemented in paper medical records) to be filled by physicians in each visit.	71 (31.4)	47 (28.7)	24 (38.7)	2.110	0.146
Q4. Add tobacco status as a vital sign (implemented in paper medical records) to be filled by physicians in each visit.	78 (34.5)	50 (30.5)	28 (45.2)	4.286	0.038

a, chi-square test

**Relationships among the knowledge, attitude, and practice scores by clusters**: the relationships between the total average scores for the KAP scores were analyzed by partial correlation coefficient controlled by age. Significant correlations were found on the scores for attitudes and practices scores of the PCPs in Cluster A (r=0.532, p<.001) and Cluster B (r=0.672, p<.001). There is no significant relationship which was found on other scores in either cluster.

## Discussion

This study's results identified two clusters exploring the demographic and KAPB patterns of PCPs towards the TDT. The findings suggest that the two clusters are different in the level of KAPB towards the TDT. The PCPs in cluster B were described by good knowledge, positive attitudes, and frequent TDT practices. This group predominantly with higher educational level, senior ranking, more males, and older. This group was labeled as the *“good knowledge, positive attitudes, and highly practices”* group. The PCPs in cluster A were different because they reported poor knowledge, lower attitudes, and fewer practicing the TDT. Compared with Cluster B, there are more females, younger with a junior ranking and had a lower educational attainment level. This cluster was identified as the *“lack of knowledge, moderate attitudes, and rarely practices”* group but comprised 72.6% of the total respondents.

The question that we might ask is why the PCPs in cluster A are lacking knowledge and rarely practices the TDT? Perhaps one of the reasons is that they acknowledged their role in advising patients to quit smoking. However, they did not accept that smoking cessation assistance is their responsibility [3,7-9]. Perhaps, they felt this is not their responsibility and barriers to providing counseling in smoking cessation because of lack of time and formal training [4]. However, the PCPCs can play an essential role in reducing tobacco use among the general population because they are knowledgeable, well trained, and willing to deliver effective tobacco intervention [6]. Many previous studies agreed that the policymakers should support the development of prevention and treatment programs for tobacco smokers actively participate in these programs with the support of physicians like the PCPs from cluster B [7-9].

The study´s results revealed a positive correlation between the attitudes and practices of the PCPs in both clusters, which suggests that the more positive their attitude, the more practices of providing TDT will be expected. This correlation is meaningful; it can help primary healthcare educators to promote this concept, especially to all junior PCPs, to create better services on TDT for their patients if needed [14,15]. Lack of knowledge appeared in cluster A which needs to be addressed by clinical-based training to improve the knowledge of the PCPs. Like the PCPs in cluster B, trained physicians could act as a mentor to advise those junior PCPS in cluster A to enhance their knowledge on TDT [8]. To date, an effective way is to modify behavior; then practice will be improved [16,17]. Therefore, tailor-make interventions for each PCP groups to improve TDT quality services are needed. The findings of this study demonstrated the importance of providing post-graduate TDT training to all PCPs too.

**Limitations**: two limitations might affect the outcomes of this survey study. First, this study was used as a self-report survey, and this will induce respondent bias that might affect the results [18]. Second, our sample was limited to one governorate of the country, so this is a limiting factor to generalize the study results.

## Conclusion

This study added new information by profiling the patterns of PCPs on their demographic, knowledge, attitudes, practice, and barriers towards TDT. Primary healthcare educators can incorporate our findings into their practice to tailor-make the content of training programs, workshops, or even other possible sympathetic forums for the target groups. However, regarding the KAPB of PCPs in TDT service and revealed the commonly associated barriers. We believe our study is just the beginning of further studies on TDT service. We hope that this study's findings could help primary health care authorities address this preventable issue and plan interventions to establish well-structured TDT clinics in the future.

### What is known about this topic

- Patients who received intensive advice from a health practitioner had a 66% and 84%, respectively, chance of quitting smoking;
- Healthcare professionals who had acquired training have been much more likely to carry out smoking cessation duties than untrained doctors;
- Smoking cessation is determined by the level of knowledge and skills of the primary care professions.

### What this study adds

- According to the knowledge, attitudes, and practices of providing tobacco dependence treatment, two groups of primary care physicians were identified. One group labeled as “good knowledge, positive attitudes and highly practices,” and the other labeled “lack of knowledge, moderate attitudes, and rarely practices” group;
- Findings could help primary health care authorities address this preventable issue and plan interventions to establish well-structured TDT clinics for people in Africa and Oman in the future.
